# Monolithic InSb nanostructure photodetectors on Si using rapid melt growth†

**DOI:** 10.1039/d2na00903j

**Published:** 2023-01-18

**Authors:** Heera Menon, Hossein Jeddi, Nicholas Paul Morgan, Anna Fontcuberta i Morral, Håkan Pettersson, Mattias Borg

**Affiliations:** a Electrical and Information Technology, Lund University Lund Sweden; b NanoLund, Lund University Box 118 Lund SE-221 00 Sweden; c School of Information Technology, Halmstad University Box 823 Halmstad SE-301 18 Sweden; d Solid State Physics, Lund University Box 118 Lund SE-221 00 Sweden; e Laboratory of Semiconductor Materials, Ecole Polytechnique Federale de Lausanne Lausanne Switzerland

## Abstract

Monolithic integration of InSb on Si could be a key enabler for future electronic and optoelectronic applications. In this work, we report the fabrication of InSb metal–semiconductor–metal photodetectors directly on Si using a CMOS-compatible process known as rapid melt growth. Fourier transform spectroscopy demonstrates a spectrally resolved photocurrent peak from a single crystalline InSb nanostructure with dimensions of 500 nm × 1.1 μm × 120 nm. Time-dependent optical characterization of a device under 1550 nm illumination indicated a stable photoresponse with responsivity of 0.50 A W^−1^ at 16 nW illumination, with a time constant in the range of milliseconds. Electron backscatter diffraction spectroscopy revealed that the single crystalline InSb nanostructures contain occasional twin defects and crystal lattice twist around the growth axis, in addition to residual strain, possibly causing the observation of a low-energy tail in the detector response extending the photosensitivity out to 10 μm wavelengths (0.12 eV) at 77 K.

## Abstract 2

In this study, we report the optical and material characteristics of an InSb nanostructure photodetector integrated directly on Si using rapid melt growth.

## Graphical abstract

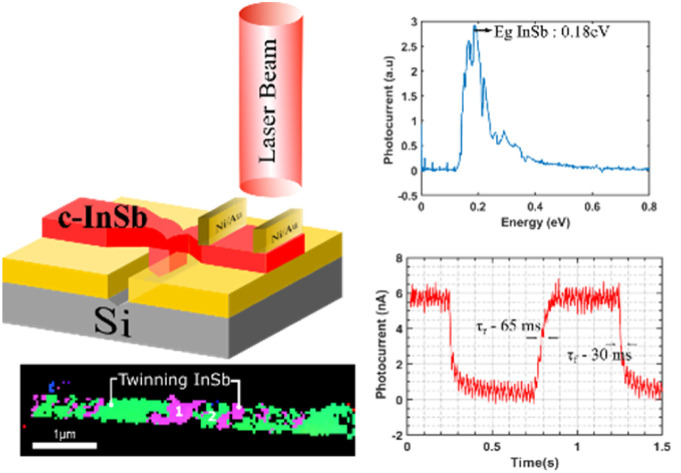

## 1 Introduction

Photodetectors in the mid-wavelength infrared (MWIR, 3–8 μm) range have for a long time been of interest for chemical and thermal detection.^1^ The MWIR spectral region gives information about environmentally important molecular trace gases such as CO_2_, H_2_O, CH_4_, NH_3_, –OH and –CH and thus could be used for atmospheric monitoring.^2^ Thermal emission from hot objects in the MWIR region can be tracked and thus has a pivotal role in military and civilian night vision applications.^3^ There is also a push to expand the operational spectrum of Si photonics from the near-infrared (NIR, 0.75–1.4 μm) to MWIR to increase the bandwidth.^4–7^

The widely used materials for MWIR photodetection are HgCdTe, InSb, and InAs/GaSb type-II super lattices (T2SLs).^8,9^ Even though HgCdTe detectors have high quantum efficiency, the material contains heavy metals, has poor stability and high cost, due to which researchers are looking into alternative materials. InSb, on the other hand, is a stable and nontoxic semiconductor, and offers higher stability as a promising alternative material for MWIR photodetectors. InSb being a direct bandgap semiconductor, with a room-temperature bandgap energy of 0.18 eV (7 μm) and a liquid nitrogen temperature bandgap of 0.23 eV (5.5 μm) could cover much of the MWIR region for photodetection.^10^ A major limitation of InSb is the lack of lattice-matched substrates, which for focal plane array detector cameras forces InSb photodetector systems to be realized by flip-chip bonding of InSb wafers onto Si CMOS read-out-integrated circuits (ROICs), which limits process accuracy, throughput and cost.^1,11^ The trend of moving towards increased pixel numbers, reduction in system size, weight and power consumption by decreasing the detector pitch sizes^12,13^ is hindered by the lack of a monolithic processes for InSb photodetectors.

Enabling monolithic integration of InSb photodetectors directly on top of the ROICs built in Si would thus be a game-changer in terms of throughput, system scalability and unit cost, but heteroepitaxy of InSb on Si is extremely challenging due to the large lattice mismatch (19.3%) and the crystal symmetry difference between the two materials. Even so, there is a considerable number of reports on InSb photodetectors on heterogeneous substrates, including on Si using thick buffer layers.^14,15^ A p–i–n InSb photodetector grown on GaAs reported a detectivity of 3.41 × 10^9^ Jones, responsivity of 1.8 A W^−1^ with a bandwidth of 8.5 GHz.^16^ Michel *et al.* reported on a InSb p–i–n photodetector on GaAs-coated Si with a detectivity of 2.8 × 10^10^ Jones.^17^ However, the use of thick buffer layers to accommodate strain is problematic in terms of the high growth temperature and long growth times, which can seriously degrade an underlying ROIC, and may not completely avoid dislocation threading into the InSb device layer. Si and GaAs are substrates that could be used for monolithic integration, and the InSb detectors reported so far either use thick buffer layers to reduce lattice-mismatch induced defects in the device layer or are separately fabricated and transferred to Si substrate. A direct integration of InSb photodetector on Si without a buffer layer has not been reported before and would reduce cost and improve precision of alignment to the ROIC. Rapid melt growth (RMG) is a technique that aids monolithic heterogeneous integration of III–V on Si. In RMG technique, an amorphous III–V film is deposited on an insulator and in contact with the Si substrate through a nanoscale opening called the seed area. The amorphous film is patterned and contained within a dielectric layer. Once the amorphous film is annealed and cooled; the melt would crystallize rapidly from the Si seed area resulting in a single crystalline material.^18,19^ In this paper, we report for the first time on InSb metal–semiconductor–metal (MSM) photodetectors, integrated without a buffer layer and with minimal thermal budget with the aid of flash lamp annealing (FLA), directly on Si substrates using the Si CMOS compatible RMG technique.^19–26^ Such a technique would enable integration of InSb photodetectors with Si CMOS circuitry at reduced price using a relatively simple fabrication process. The optical characterization of the photodetector is reported and is supported by an in-depth investigation of the crystallographic properties of the InSb nanostructures and its effects on performance of the photodetector.

## 2 Experimental details

**Fig. 1:**
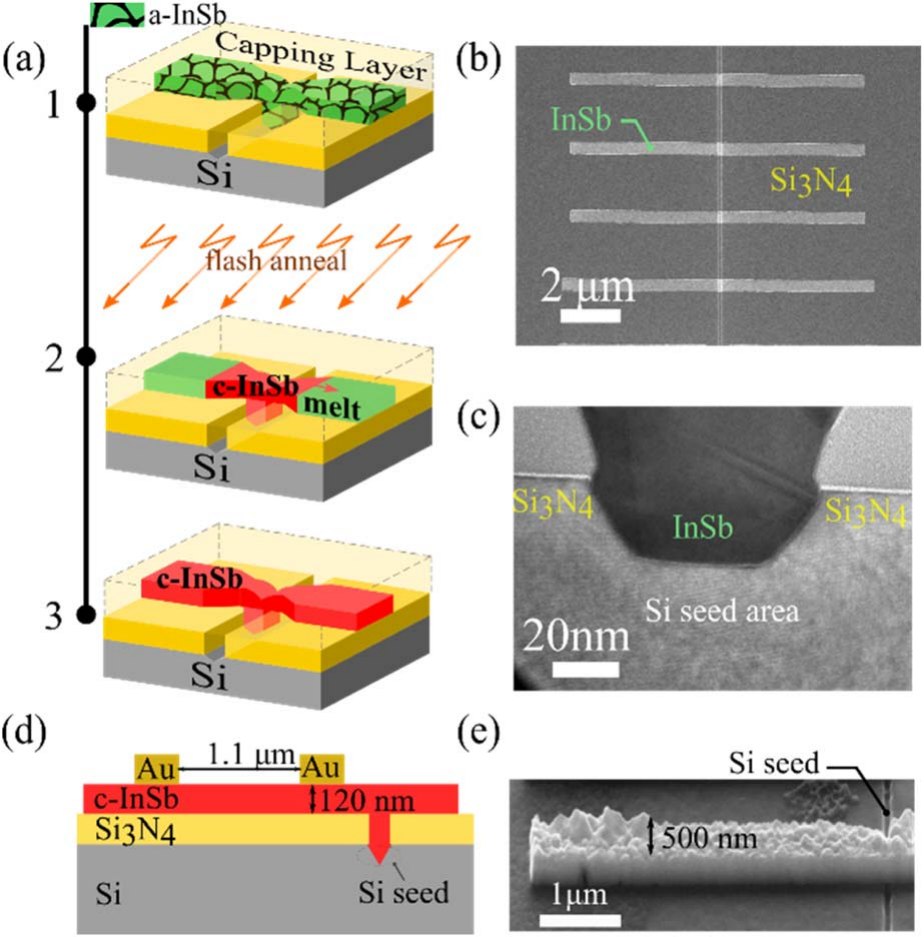
(a) Schematic overview of the RMG process. (1) Patterned amorphous InSb, in contact with the Si seed area and capped with a dielectric layer. (2) Flash annealing of the sample causing melting and rapid recrystallization of InSb starting from the seed area. (3) Schematic of the resulting crystalline InSb structure. (b) SEM image of the seed area and the InSb nanostructures before crystallization. (c) TEM cross sectional view of the Si seed area and InSb. (d) Schematic of the InSb detector (e) SEM image showing the InSb nanostructure used to make the photodetector.

The fabrication procedure was previously reported in detail.^20,21^ The overview of the fabrication process is illustrated through the schematic diagram in Fig. 1(a). The fabrication process is initiated with the deposition of 40 nm Si_3_N_4_ on a Si (100) 3-inch wafer by means of inductively coupled plasma chemical vapor deposition (ICPCVD). After the deposition of the bottom dielectric, an opening into the Si substrate (also referred to as the seed area) is formed using electron beam lithography and dry etching of Si_3_N_4_. The Si seed area would act as the starting point for the InSb crystal growth and may provide a crystalline template for epitaxial growth. This step is followed by a partial V groove formation in the Si seed area using TMAH (25%) etch at 60 °C. After the creation of the V groove, the sample was etched using HF (1%) for 10 s to ensure that the native oxide in the seed areas is removed. Then the sample was loaded into the MBE chamber for the deposition of 120 nm of amorphous InSb at 240 °C. The resulting layer following MBE is slightly In rich (51.5% In, 48.5% Sb). Following this, the InSb was patterned into structures overlapping with the seed areas (Fig. 1(b)) of 5–10 μm length and with widths ranging from 200 nm to 1000 nm using EBL and CH_4_-based reactive ion etching. Once the InSb structures were patterned, the sample was capped with a layer of 13 nm Al_2_O_3_ and 950 nm Si_3_N_4_ (also referred to as the crucible). The sample was then heated up to 335 °C in vacuum and flashed with a 1.5 ms pulse of white light with 19.5 J cm^−2^ energy density to briefly reach a peak temperature just above melting of InSb (525 °C), making the full process within the thermal budget of CMOS processing.^27^ The crucible contains the material during the short period in the melted state. Upon cooling, the undercooled melt drives the crystallization of InSb from the Si seed area upwards and then to two directions (left and right) creating two growth fronts. In cool down phase, the temperature drops below 400 °C in the time scale of milliseconds and thus decomposition of InSb at this stage is minimal. After the annealing and cooling phase, the Si_3_N_4_ capping layer was removed using SF_6_ based reactive ion etching, allowing for visual inspection of InSb nanostructures. Occasionally, voids and gaps in the InSb structures are observed, caused by formation of In droplets in the heating step before the flash, leading to In diffusion into the surrounding layers. This issue could in the future be avoided by tighter control of the stoichiometry of the deposited InSb film.

The recrystallized InSb nanostructures were characterized using electron back scatter diffraction (EBSD) and scanning electron microscope (SEM). The EBSD images were further analyzed using the MTex toolbox in Matlab.

To fabricate single nanostructure based InSb photodetectors, the recrystallized InSb nanostructures were contacted by Ni/Au electrodes formed in a lift-off process. The electrode separation is 1.1 μm with a device width of 500 nm. It is expected that an increase in width and decrease in length of the nanostructure could increase the photogenerated current. However, in this work we focus on a single device geometry. Studies have shown that GaSb nanostructures protected from oxidation using an oxide template resulted in higher mobility.^28^ Therefore, the Al_2_O_3_ capping layer was only removed in the contacted regions before metallization, leaving it to protect the InSb from oxidation elsewhere as oxidized InSb surfaces are known to contain a high density of defect states.^29,30^ Spectrally resolved photocurrent (PC) measurements were carried out at 77 K using Fourier transform infrared (FTIR) spectroscopy using a built-in globar IR source and KBr beam-splitter. We also recorded the time-response of the photodetector at 1550 nm by modulating the laser with a 1 Hz square wave using a pulse generator combined with a storage oscilloscope at different laser power.

Micro-Raman spectroscopy was performed using a Renishaw inVia Raman microscope with a 532 nm laser in a backscattering, unpolarized configuration. The laser was focused on the nanostructures with a microscope objective with numerical aperture NA = 0.85 and detected using a 3000 L mm^−1^ diffraction grating and a CCD camera. Reference spectra were collected from InSb (110) and (100) bulk crystal faces to establish the expected bulk TO and LO peak positions, respectively.

## 3 Results and discussion

### 3.1 Photoresponse

**Fig. 2:**
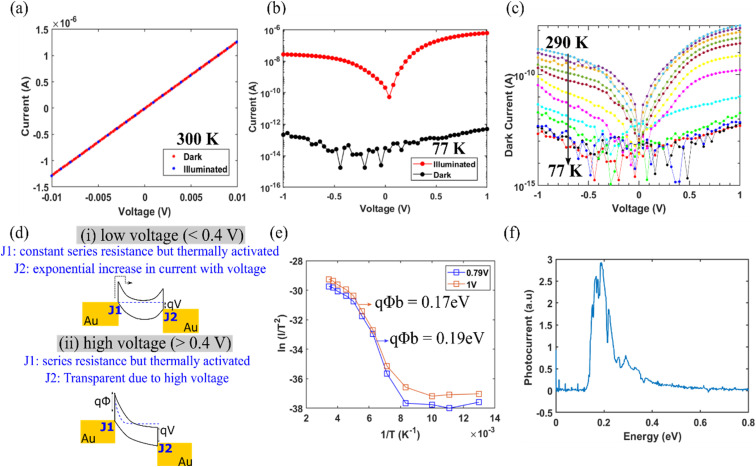
Current–voltage (*I*–*V*) curves of a single InSb nanostructure photodetector in dark and under illumination with a globar IR source at (a) 300 K and (b) 77 K. (c) Temperature-dependent *I*–*V* traces in dark. (d) Energy band diagram of the photodetector with indicated metal–semiconductor contacts. (e) Extraction of the Schottky barrier height at two different applied biases from the data in (c). (f) Spectrally resolved photocurrent.

Fig. 2(a) presents the *I*–*V* characteristics of the MSM photodetector at room temperature (RT), where an ohmic behavior with no measurable difference between dark and illuminated conditions is observed. This is to be expected considering the narrow band gap of InSb, for which thermal carrier excitation will dominate the current transport at RT. From the known work function of Ni (5.25 V) and smaller electron affinity of InSb (4.59 eV) we can, in a first approximation, assume that a significant Schottky barrier should be present at the Ni–InSb junction, although the precise barrier height depends on the details of how the interface forms. By cooling the photodetectors to 77 K, thermal excitation is suppressed and indeed Schottky barriers at both contacts cause nonlinear *I*–*V* characteristics for which the current increases exponentially at low bias while at high bias the device responds in an ohmic fashion. Under these conditions we observe a clear response to illumination, with a difference of more than 2 orders of magnitude between the dark current and PC for positive biases (Fig. 2(b)). At high biases >0.4 V the forward-biased junction (J2) will be transparent due to the high voltage, while the reverse-biased junction (J1) will act as a constant series resistance set by the Schottky barrier height (Fig. 2(d)). Thus, by evaluating the temperature dependence of the *I*–*V* characteristics at large biases we can estimate the Schottky barrier height. The dark current for different temperatures is shown in Fig. 2(c). The dark current measured at 1 V falls by 3 orders of magnitude from 16.5 nA at 290 K to 10.6 pA at 140 K, after which the dark current stabilizes at 120 K and below. The barrier height of the metal–semiconductor contact was estimated using the Schottky diode equation in the following logarithmic form:1
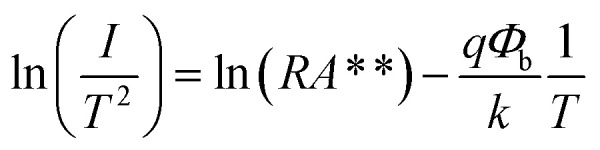
where *qΦ*_b_ is the activation barrier for thermal carrier injection, *R* is the electrically active area, A** is the Richardson constant, and *I* is the measured current.^31^ For a fixed external voltage V, the plot ln(*I*/*T*^2^) *versus* 1/*T* yields the barrier height *Φ*_b_ from the slope of the curve as shown in Fig. 2(e). For the temperature range of 120 K to 200 K, we extract a *qΦ*_b_ of around 0.17 eV to 0.19 eV, which is close to the size of the band gap. As expected, we obtain a constant barrier height for forward biases above 0.4 V (Fig. S1 in ESI†). Previously, Fermi level pinning at the valence band edge was observed in n-type InSb, giving rise to a sizeable surface electric field under dark conditions.^32^ Van der Pauw and Hall measurements on RMG InSb (Fig. S2 in ESI†) confirms that the material is n-type like in our previous work^33^ and we thus expect the band structure to resemble the schematic in Fig. 2(d), where the Fermi level is pinned close to the valence band edge creating a barrier that blocks transport to the electrodes when there is no illumination.

Next, FTIR PC spectroscopy was carried out to investigate the photoresponse of the InSb nanostructure photodetectors at 77 K. Fig. 2(f) shows the spectrally resolved PC. The expected bandgap of InSb at 77 K is around 0.23 eV and we do observe a peak in PC close to this energy (0.18 eV). However, the PC onset is redshifted to around 0.12 eV, thus significantly extending the photoresponsivity of the device out to 10 μm. The reason for this could be a bandgap narrowing induced by stress formed during the rapid crystallization of InSb from the melt in the RMG process, or the result of defect states near the conduction band edge.^34^ This is further investigated using EBSD in Section 3.3.

### 3.2. Photosensitivity

**Fig. 3:**
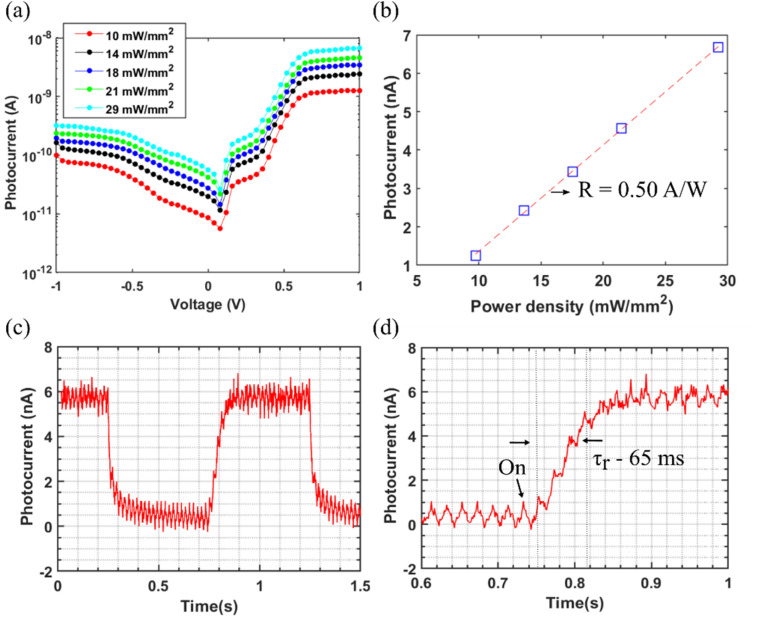
(a) *I*–*V* curves under 1550 nm illumination with different power densities at 77 K. Here, for instance the power density of 29 mW mm^−2^ corresponds to 16 nW power on a single photodetector element. (b) Dependence of PC on power density at 1 V bias. The slope of the plot corresponds to responsivity with consideration of device area. (c) Time-resolved PC response to a 1550 nm laser pulse train. (d) Time-resolved PC onset for the extraction of rise time.

To explore the photosensitivity of the InSb MSM at 77 K, the device was illuminated with a calibrated 1550 nm diode laser. Fig. 3(a) presents the *I*–*V* characteristics for different laser power densities. For our measurements, the laser's optical output at a certain current was first measured using a power meter, while the power meter was covered with a cap with 1 mm^2^ hole in it. In this way we correlate the measured laser currents to optical power density (mW mm^−2^). As an example, a 100 mA corresponded to a power density of 19.5 mW mm^−2^. The distance between laser and the sample/power meter was kept constant throughout the experiments (measurement setup is shown in Fig. S3 in ESI†). The PC (*I*_ph_), defined as the increase in current under illumination, grows linearly with increasing laser power (Fig. 3(b)) over the investigated power density range. This allowed us to reliably extract the device responsivity from the measurement results. Responsivity (*R*) is the amount of output PC per incident optical power *i.e.*,2
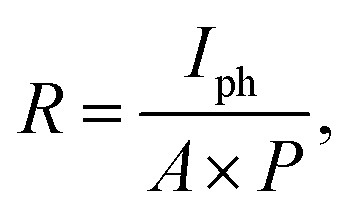
where *A* is the active area of the detector (0.5 × 1.1 μm^2^) and *P* is the laser power density.^35,36^

In this case, the responsivity extracted from the slope of Fig. 3(b) is 0.50 A W^−1^. Here we note that 29 mW mm^−2^ laser power density corresponds to 16 nW incident power onto the device. The PC variation with power density for different biases, as well as the variation of responsivity with bias, are given in the Fig. S4 and S5 in ESI.† Worth noting is that for all positive biases the responsivity remains nearly constant with varied illumination power, in contrast to many nanoscale photodetectors which report their highest responsivity values for ultralow power only. The previously reported single nanowire InSb photodetectors are of horizontal configuration in which the nanowires are transferred from the free-standing position to a lying position on another substrate horizontally.^37^ An InSb MSM photodetector fabricated using electrochemically synthesized InSb nanowires has been reported to have a responsivity of 8.4 × 10^4^ A W^−1^ (ref. 38) for an incident light at 5.5 μm at 0.49 mW cm^−2^. Our work involves direct integration of InSb detector on Si substrates, which has not been previously reported. Previous work on GaSb photodetectors fabricated using the RMG technique has reported a responsivity of 0.57 A W^−1^ for a power of 50 μW.^39^ A responsivity of 311 A W^−1^ at 4.3 μm for incident power of 1 pW was reported for an InSb nanosheet photodetector, while the responsivity at 100 pW was about ten times lower.^35^ Given our higher incident power (5–16 nW), the obtained responsivity values are thus reasonable. One limiting factor is the small device thickness (120 nm) which only absorbs about 20% of the incoming radiation at 1550 nm, estimated from the known absorption coefficient of InSb.^40^ In addition, we estimate that up to 40% of the incoming light could be reflected at the top surface, while reflection at the back InSb surface has only a marginal effect on responsivity (<10%). Future work may thus effectively optimize responsivity by engineered reflective coatings above and below the active InSb layer. Another limiting circumstance in the present case is that we do not have access to any quantum cascade laser operating at the bandgap of around 0.2 eV. By normalizing the spectrally resolved PC in Fig. 2(f) to optical power and using the know responsivity of 0.50 A W^−1^ at 1550 nm, we can estimate lower boundary of about 5 A W^−1^ at 6.1 μm.

Finally, we measure the response time of the photodetector using time-resolved PC measurements. Fig. 3(c) and (d) show the transient photoresponse under illumination with a modulated (1 Hz) 1550 nm diode laser @ 16 nW and 1 V bias. The rise and fall time of the detector is around 65 ms and 30 ms and is similar to or shorter than the reported values of the InSb nanosheet and InAs nanosheet photodetectors, which range from seconds to milliseconds.^38,41,42^ We do not observe response time in the nanosecond time scale as expected from diffusive transport transit time which can be explained by the large surface-to-volume ratio of our ultrathin device, causing delay of the photocurrent response through capture-emission processes *via* surface trap states. Although the intrinsic protection of the InSb surface is expected to provide some degree of surface passivation,^28,43,44^ further work will be needed to optimize the response speed of the photodetectors. In summary, our first experimental results for monolithically integrated InSb MSM photodetectors presented here indicate high responsivity in the MWIR spectrum (peak at 6.1 μm) and a relatively fast response speed and are thus promising for further work in this direction.

### 3.3. Crystallographic analysis

For monolithic InSb photodetectors formed by the RMG method to be a viable approach, we need to properly understand the crystallographic features that arise from the RMG process. Since we have observed a redshift in the spectrally resolved PC (Fig. 2(f)), indicating band gap narrowing that may be due to strain and/or crystal defects, we have invoked EBSD to carefully analyze the local crystalline properties of the InSb structures after the RMG process.

#### 3.3.1. Twin defect formation

**Fig. 4:**
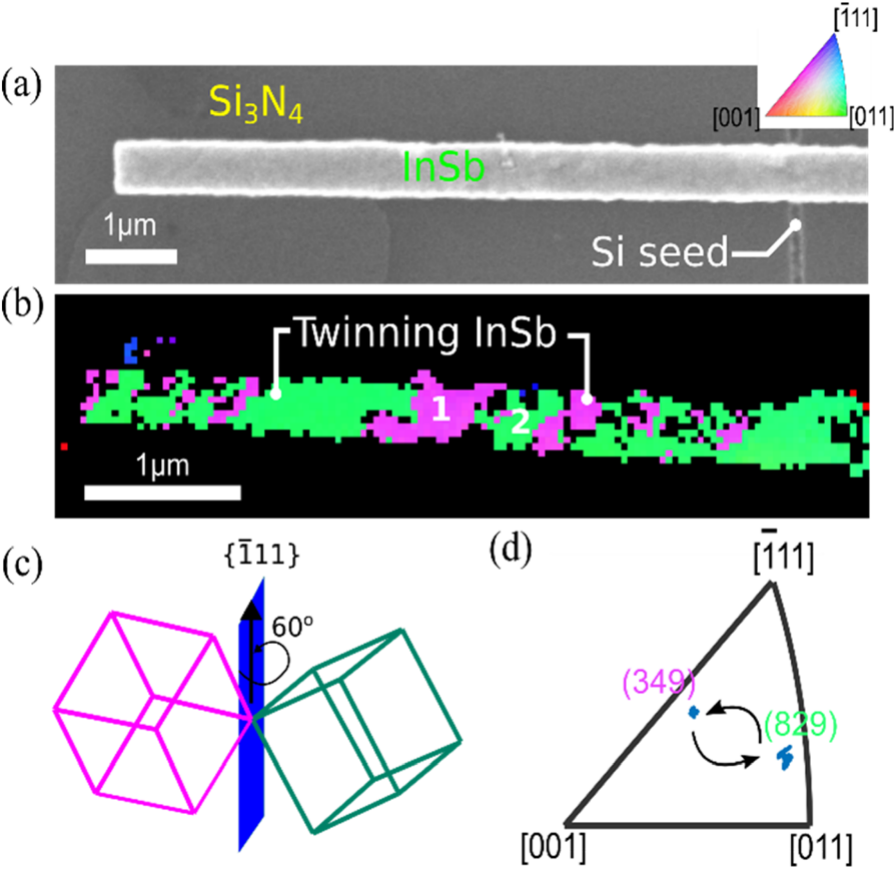
(a) SEM image of the analyzed InSb structure. (b) EBSD Z-orientation map of the InSb structure revealing repeated twin formation in the InSb crystal. 1 and 2 represents the domains used for the analysis. (c) Schematic representation of crystal rotation around [1̄11] axis (d) inverse pole figure showing the two twinned domains.

One interesting crystallographic feature that is revealed in some InSb structures by the EBSD orientation maps is occasional and repeated twin defects. Fig. 4(b) shows a typical such structure with a clearly repeated pattern of two similar colors. Such defects, although prominent in our measurements, should have minor effects on the electrical and optical properties of the semiconductor,^45,46^ but it is important to verify that our observations indeed correspond to twinned domains and not separate crystalline grains. Twin plane defects in III–V semiconductors correspond to a rotation of the crystal lattice by 60° around a [1̄11] direction. Therefore, we carefully analyze the domain orientations relative to each other. Having determined the mean orientation of the two types of domains, [349] (pink) and [829] (green), respectively, the first (pink) domain orientation is transformed by 60° rotation around the [1̄11] axis. The stereographic projections of the transformed pink and original green lattices are presented in the Fig. S6 in ESI,† and perfectly coincides. This confirms the presence of discrete twin defects in the InSb structures. Twin defect formation is often observed in nanostructured growth of III–V semiconductor and is related to growth conditions variations like temperature instabilities, presence of impurities or morphological instability at the crystallization front.^47–49^ In-containing III–V semiconductors are sensitive to twin formation due to the relatively higher ionicity of the atomic bonds,^50–52^ while of these, InSb is least sensitive. In our work, the rather rough top surface of the crucible could disturb the growth front and contribute to the twin formation.

#### 3.3.2. Crystal lattice rotation

**Fig. 5:**
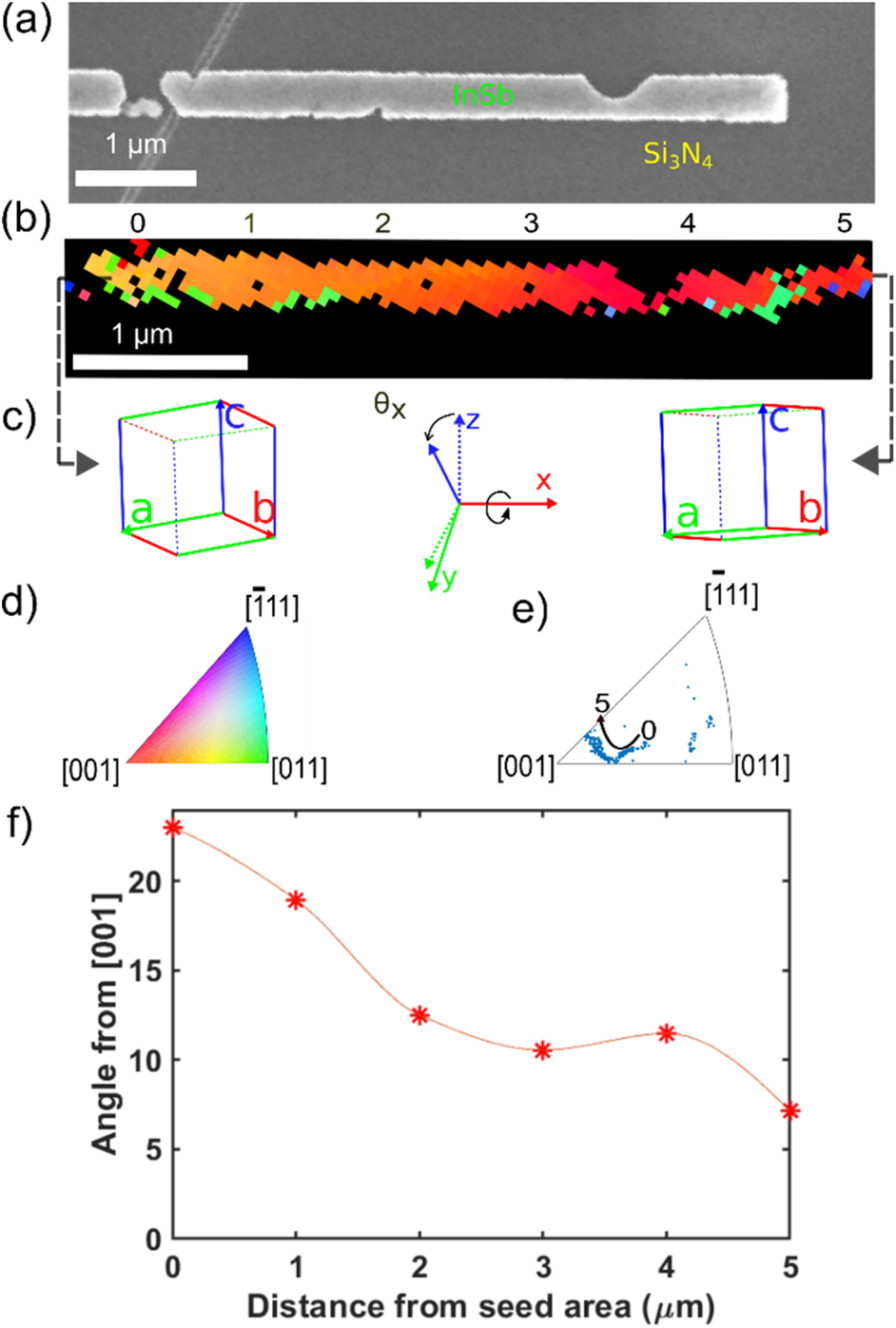
(a) SEM image of a flash annealed InSb nanostructure after removal of the capping layer. (b) The EBSD Z orientation of the InSb structure and (c) schematic representation of the crystal orientation at the beginning of the seed area and at the end of the seed area with the indication of rotation direction. (d) Colour-code for EBSD orientation map. (e) Inverse pole figure showing the lattice rotation. (f) Angular difference of different orientations of the InSb crystal with the (001) plane as a function of distance from Si seed area.

Another interesting crystallographic feature pertinent to recrystallized InSb nanostructures is crystal lattice rotation. Here, we investigate the evolution of the crystalline orientation as a function of the distance away from the seed area, where the InSb crystal is nucleated from the InSb melt and rapidly grows outward during the recrystallization process. Previous studies on Ge and SiGe RMG reports significant lattice rotation away from the seed area.^53–58^ We collected crystallographic orientation data for single InSb structures, using an electron beam scanned with a step size ranging from 40 to 80 nm, in many cases confirming the presence of crystal lattice rotation. Fig. 5(b) illustrates such an EBSD Z-orientation map of an InSb nanostructure with a gradual change in orientation. It could be observed that the crystal near the seed area has an orientation near [012], which rotates significantly around the horizontal axis parallel to the crystallization direction over the 5 μm long InSb structure (*θ*_*x*_ in Fig. 5(c)), leading to a crystal twist along the structure. The rotation of the crystal is also represented in an inverse pole figure (Fig. 5(e)), which clearly shows how the crystal orientation change progresses. The crystal lattice rotation phenomenon was studied as a function of distance from the Si seed area by analyzing the EBSD map. Fig. 5(f) summarizes the angular difference of each point in the InSb crystal from the (001) plane of the Si substrate, initially being close to [012] orientation and rotating as much as 15° towards the [001] direction over the 5 μm length of the structure. There are several possible driving forces for the lattice rotations. Toko *et al.*^56^ reported that the minimization of interfacial energy between the Ge and the insulator was the key contributor to the lattice rotation in RMG growth. In addition, an influence of heating too far above the melting point and cooling rate on the magnitude of lattice rotation has also been reported.^53,58^ This effect could be related to the latent heat of solidification released at the solid–liquid interface during the rapid crystallization. It has been noted for Ge RMG that the latent heat in the growth front can be more efficiently conducted away by the solid crystal on one side than the liquid on the other,^53,59–61^ as the thermal conductivity for the Ge solid is higher than for the liquid. The same situation applies to InSb, for which the thermal conductivity of crystalline InSb (17.7 W mK^−1^)^62^ is almost twice that of liquid InSb (9.23 W mK^−1^).^63^ In this case, the locally strong temperature gradient at the interface combined with very high rates of crystallization can thus be a driving force for lattice-rotation. The often strong crystal twist found in our InSb nanostructure likely causes residual strain that can explain the observed bandgap narrowing in our photodetector.^64^

#### 3.3.3. Epitaxial relationship

We finally investigate the crystalline relationship between the InSb-OI microstructures and the Si substrate. By EBSD we map the crystalline orientation of 55 InSb single nanostructure crystals close to the seed area to determine whether there exists an epitaxial relationship between the InSb and Si despite the very large lattice-mismatch.

**Fig. 6:**
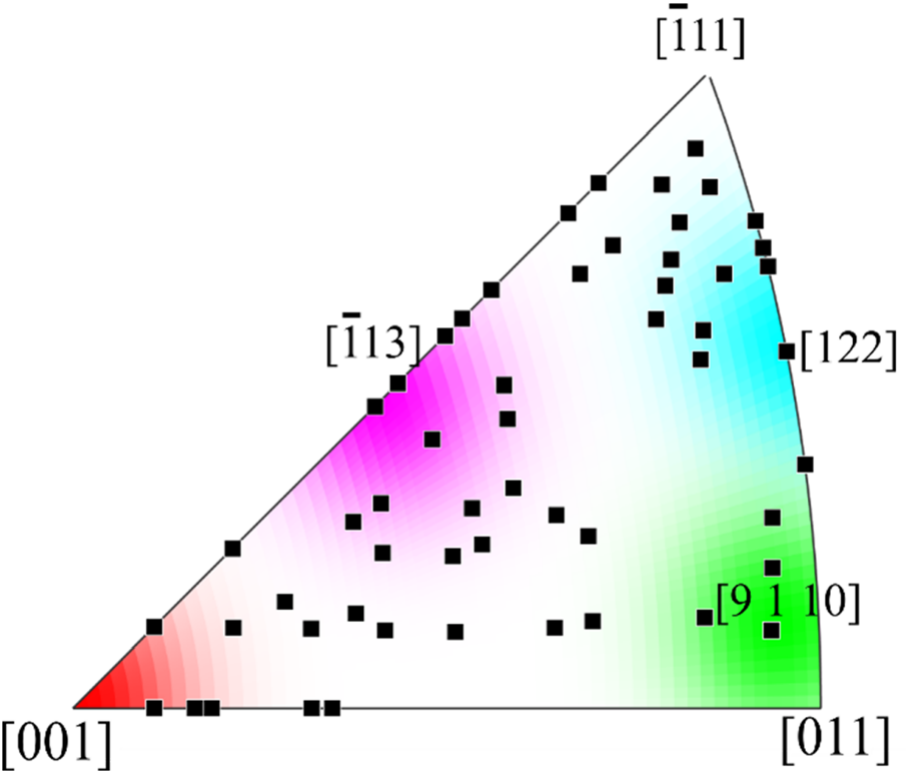
Orientation of InSb above the seed area extracted from EBSD Z orientation map for 55 structures and plotted on the inverse pole figure. Each orientation is associated with a specific color in the inverse pole figure. Here, few orientations are emphasized with the color coding extending to the vicinity of 5°.

The collected statistics on the out-of-plane InSb crystal orientations are presented in the inverse pole figure (IPF) in Fig. 6. Four specific crystallographic orientations [001], [113], [9 1 10], and [122] orientations are indicated with a specific color in the IPF map, for reasons that will be explained in the following. The intensity of the color extends to 5° outside the specific orientation. Here we can see that there are data points close to the [001] orientation (red in Fig. 6), which is same as the substrate, indicating that some nanostructures have a direct epitaxial relationship. The large lattice-mismatch between InSb and Si leads to the formation of dislocations at the heterojunction, and an inhomogeneous strain field at the heterointerface could thus easily cause a few degrees of crystal lattice tilt,^65^ which accounts for the spread of the data points around the [001] orientation. In addition, some degree of lattice rotation may already occur close to the seed area.

As we previously concluded that twin defects are often present in the InSb nanostructures, we should also see epitaxial but twinned domains having orientations close to [122] (cyan in Fig. 6). Indeed, we do see a clustering of data points close to this orientation.

However, the most prominent cluster of data rather has an orientation close to [113]. Previously, heteroepitaxial InSb films on Si (111) substrates were reported to be 30° rotated^66^ around the (111) axis which reduced the 19% lattice mismatch with Si to 3.3%.^67^ A similar study of InSb on V-groove Si (001) also reported the same 30° rotation of InSb films on <111> facets of the V-groove.^68^ Interestingly, rotation of [001] around *a* <111> axis at an angle of 30° yields <1 1.3 3.7> which is close to [113], suggesting that a similar effect could be present in our present work.

Of course, twinning of these rotated structures may also occur, which would result in orientations close to [9 1 10]. We can confirm the presence of structures with orientations close to this direction, shown as green in Fig. 6. In conclusion, we thus observe a complicated but epitaxial relationship between the crystal orientation of the InSb nanostructures and the Si substrate, the spread of the data indicating considerable residual strain. In addition, some data points fall outside this categorization, and we cannot exclude that some structures have grown in a non-epitaxial manner.

The variations in crystal orientation induced by the initial nucleation, twinning and lattice twist are not expected to have a direct effect on the photocurrent response of the InSb photodetectors, as the band structure of InSb is direct and symmetric around the *Γ*-point. However, an indirect effect could be that high-index facets on the top and bottom interface of the device may have higher surface recombination velocities than low index (001) or (110) surfaces.^69^ To evaluate this would be a topic for future studies.

#### 3.3.4. Raman spectroscopy

**Fig. 7:**
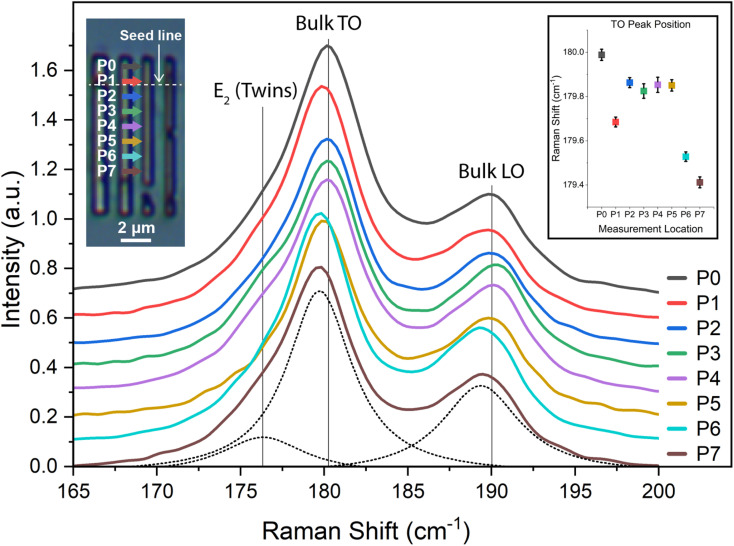
Raman spectra taken along a line scan of a 500 nm-wide InSb nanostructure. The top left optical micrograph inset shows the measurement locations. Vertical lines show the positions of the TO and LO Raman peaks as measured from a bulk InSb reference. The *E*_2_ peak is also indicated, which appears for wurtzite-like crystal structures, indicating the presence of twins. The spectrum for P7 is fitted to all three peaks, as shown by the dotted lines. The top right inset shows the fitted TO peak position and corresponding standard error at every point along the line scan.

To complement the crystallographic findings presented above, Raman spectroscopy was performed on select InSb nanostructures. The measurements were performed in a confocal microscope in the backscattering configuration under 532 nm excitation. The spot size is below 1 μm. Fig. 7 presents a series of Raman spectra taken along the length of a 500 nm-wide InSb strip, as indicated in the top left optical micrograph inset. The spectra are characterized by the presence of the transversal optical (TO) and longitudinal optical (LO) modes. The reference value of the peaks is indicated with a vertical line on the graph. The intensity and lineshape of the peaks give insight into the crystal orientation and quality of the InSb. For example, one would expect to observe predominantly the LO mode in the (100) orientation. The appearance of both the LO and TO is consistent with the mixture of orientations and preponderance of the (111) orientation. The observed TO peaks exhibit a slight asymmetry, with the appearance of a shoulder toward lower wavenumbers. This spectral feature is even more pronounced in spectra from other nanostructures, not shown here. We attribute this shoulder to the presence of the *E*_2_ peak, associated with the presence of wurtzite phase and/or a high density of twinning defects.^70^ The inset in Fig. 7 illustrates the shift of the TO peak along the structure. The deviation of the TO peaks from the measured bulk TO position indicates varying amounts of tensile strain. The largest shift comes from position 7, with a peak shift of 0.85 cm^−1^, which would correspond to ∼0.11% strain, or 167 MPa of hydrostatic pressure.^71^ The strain does not strictly increase along the length of the structure, and some regions appear nearly relaxed. To conclude, the findings from this Raman study are coherent with our conclusion on the presence of tensile strain and twinning in these nanostructures and could explain the observed red shift of the optical absorption edge.

## 4. Conclusion

In conclusion, we have fabricated MSM single nanostructure InSb photodetectors monolithically integrated on Si using the RMG technique and analyzed their electrical and optical characteristics, together with their crystallographic properties at length. *I*–*V* measurements of the device at 77 K in dark and under illuminated conditions indicated more than 2 orders of magnitude difference in the photocurrent. Time-resolved photocurrent measurement using 1550 nm excitation demonstrated a response time of milliseconds, and a responsivity of 0.50 A W^−1^ at 16 nW, with extrapolated responsivity of 5 A W^−1^ at 6.1 μm wavelength. Spectrally resolved photocurrent measurements indicated a photocurrent onset below (0.12 eV) the expected bandgap of InSb (0.23 eV). This extends the photosensitivity of the device out to 10 μm and to find the origin of this led to further investigation of the crystallographic features formed in the single crystalline InSb during the RMG process. EBSD analysis of the InSb structures identified significant crystal twist, twins and evidence of local strain fields, which can explain the red shift of the photoresponse. The dominating crystal orientations indicate an epitaxial relationship to the Si substrate in most cases, which is complicated by the indications of crystal rotation at the onset of growth as well as occasional twinning. The presented results give an important insight into the potential and needs for further optimization to enable monolithically integrated InSb photodetectors on Si.

## Conflicts of interest

There are no conflicts to declare.

## Supplementary Material

NA-005-D2NA00903J-s001
