# Supplementary material for: Monolithic InSb nanostructure photodetectors on Si using rapid melt growth
Source: Nanoscale Adv. 2023 Jan 18;5(4):1152–62. doi: 10.1039/d2na00903j (PMC9926903; doi:10.1039/d2na00903j)
Supplement: NA-005-D2NA00903J-s001 [file NA-005-D2NA00903J-s001.pdf]

Electronic Supplementary Material (ESI) for Nanoscale Advances.
This journal is © The Royal Society of Chemistry 2023

Supplementary Material

Monolithic InSb nanostructure photodetectors on Si using Rapid Melt Growth

Heera Menon[1,2], Hossein Jeddi[2,3,4], Nicholas Paul Morgan[5],

Anna Fontcuberta i Morral[5], Håkan Pettersson[2,3,4], Mattias Borg[1,2]

1. Electrical and Information Technology, Lund University, Lund, Sweden

2. NanoLund, Lund University, Box 118, SE-221 00 Lund, Sweden

3. School of Information Technology, Halmstad University, Box 823, SE-301 18 Halmstad, Sweden.

4. Solid State Physics, Lund University, Box 118, SE-221 00 Lund, Sweden.

5. Laboratory of Semiconductor Materials, Ecole Polytechnique Federale de Lausanne, Lausanne, Switzerland

Schottky barrier height variation with applied forward bias

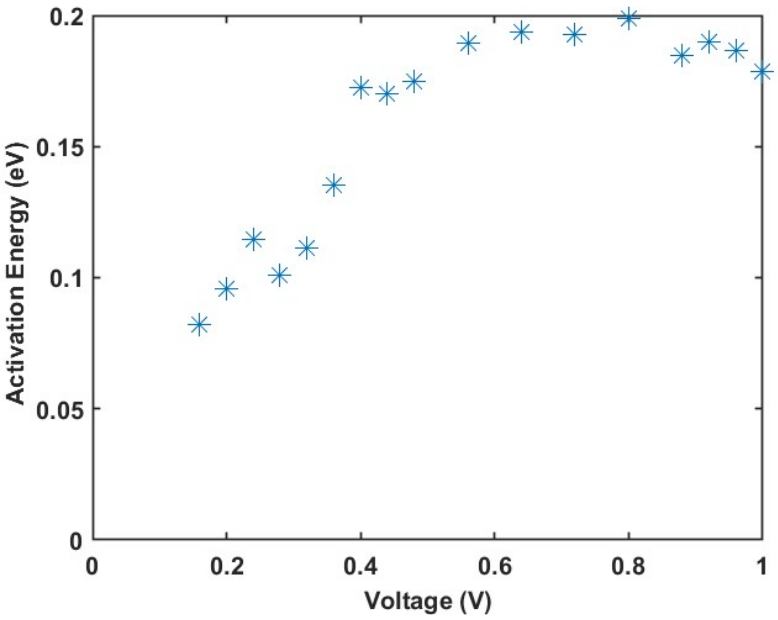

Figure S1. Plot showing the variation of activation energy with applied forward bias.

Hall Measurement of InSb devices

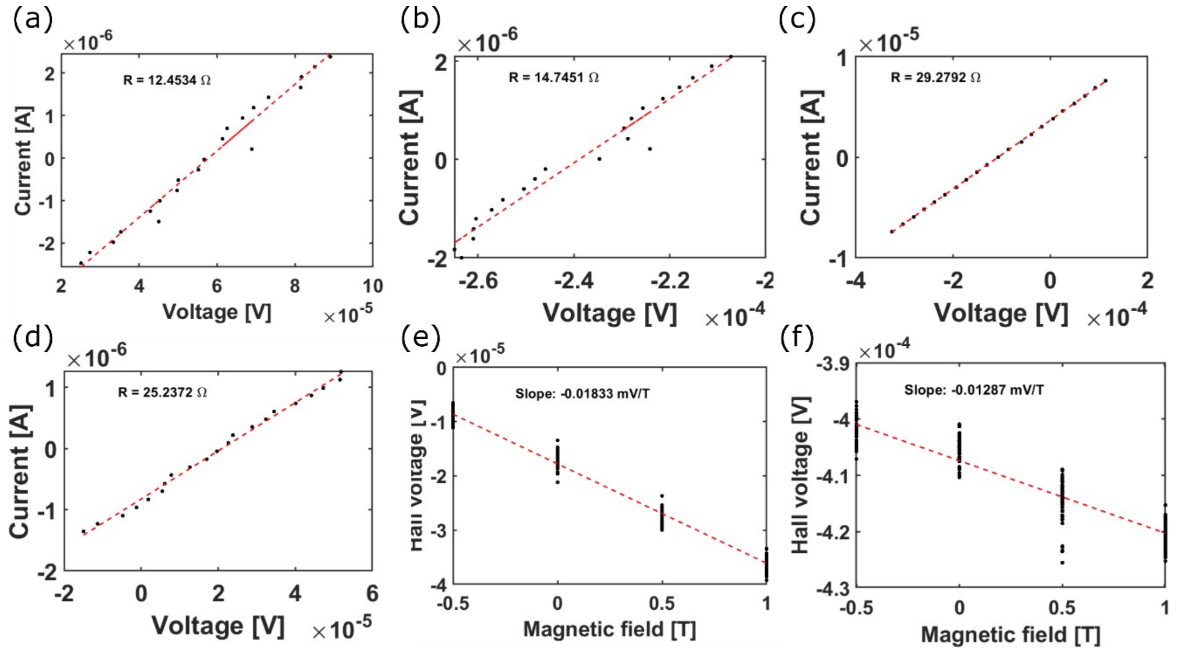

Figure S2: Raw data of Hall measurement on InSb device. (a-d) shows the Van der Pauw measurement results between four contacts and (e-f) shows the plot of Hall voltage with varying magnetic field. The negative slope of the Hall voltage indicates n-type conduction.

Experimental Set-up

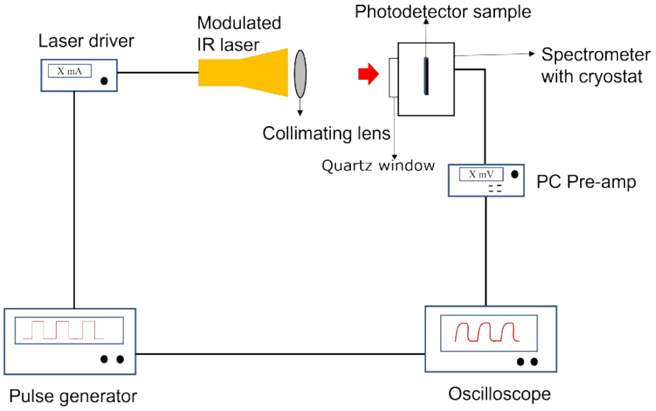

Figure S3 IR laser excitation setup. The modulated laser beam passes through three quartz windows (each quartz window has 92% transmission) located in front of the sample.

Responsivity of InSb photodetector

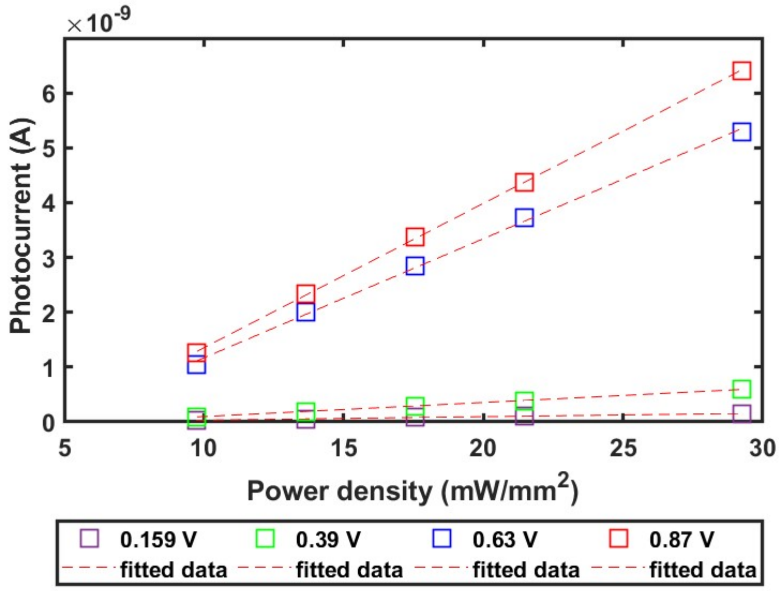

Figure S4 Photocurrent obtained for different power densities at specific voltages.

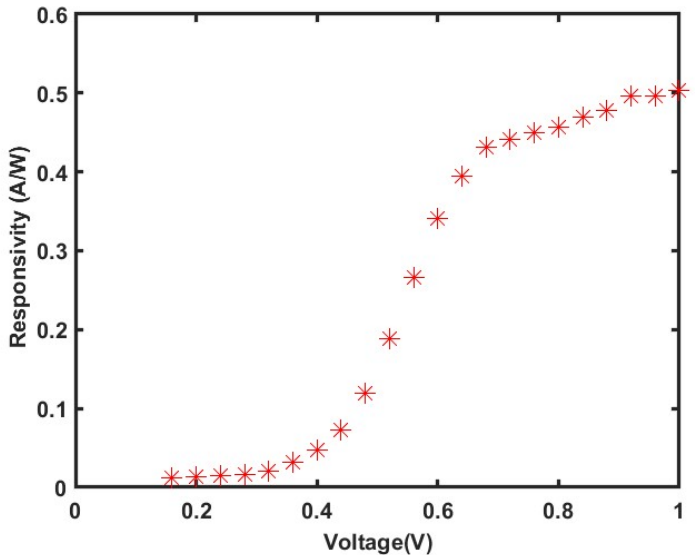

Figure S5 Variation of responsivity with bias

Twinning misorientation

To check whether two grains are related by twinning misorientation, the following procedure is used in MTex:

1. The mean orientation of the two grains is extracted.
2. The reference frame of one crystal coordinate is transferred to the other crystal reference frame. This transformation is referred to as misorientation.
3. The stereographic projection of major lattice planes of one crystal reference plane and the transformed reference plane are plotted together. If there is a perfect coincidence between these lattice planes this could indicate a presence of twinning.
4. We now define a misorientation between the perfectly coincident plane and extract the rotational axis and the angle of rotation. In our study we have observed the rotational axis to be [-1 1 1] with a rotational angle of 60°, as expected for twin plane defects.

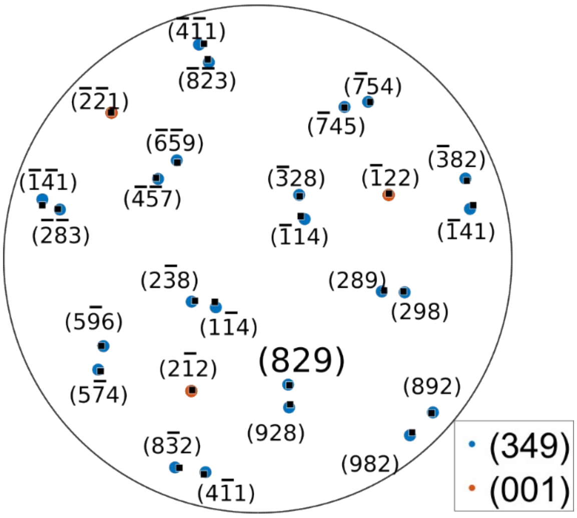

Figure S6 Stereographic projection showing coincidence between the lattice planes of two crystals. There is coincidence between (001) and (-122) as well as (349) and (829), thus indicating that these orientations could be twinning disorientation.
